# Evidence of strong stabilizing effects on the evolution of boreoeutherian (Mammalia) dental proportions

**DOI:** 10.1002/ece3.5309

**Published:** 2019-06-14

**Authors:** Tesla A. Monson, Jean‐Renaud Boisserie, Marianne F. Brasil, Selene M. Clay, Rena Dvoretzky, Shruti Ravindramurthy, Christopher A. Schmitt, Antoine Souron, Risa Takenaka, Peter S. Ungar, Sunwoo Yoo, Michael Zhou, Madeleine E. Zuercher, Leslea J. Hlusko

**Affiliations:** ^1^ Department of Integrative Biology University of California Berkeley California; ^2^ Human Evolution Research Center University of California Berkeley California; ^3^ Museum of Vertebrate Zoology University of California Berkeley California; ^4^ Anthropologisches Institut und Museum Universität Zürich Zürich Switzerland; ^5^ PALEVOPRIM, CNRS & Université de Poitiers POITIERS Cedex 9 France; ^6^ Department of Human Genetics University of Chicago Chicago Illinois; ^7^ Department of Anthropology and Biology Boston University Boston Massachusetts; ^8^ UMR 5199 PACEA Université de Bordeaux PESSAC France; ^9^ Department of Anthropology University of Arkansas Fayetteville Arkansas

**Keywords:** Boreoeutheria, dentition, diet, life history, phylogenetic signal

## Abstract

The dentition is an extremely important organ in mammals with variation in timing and sequence of eruption, crown morphology, and tooth size enabling a range of behavioral, dietary, and functional adaptations across the class. Within this suite of variable mammalian dental phenotypes, relative sizes of teeth reflect variation in the underlying genetic and developmental mechanisms. Two ratios of postcanine tooth lengths capture the relative size of premolars to molars (premolar–molar module, PMM), and among the three molars (molar module component, MMC), and are known to be heritable, independent of body size, and to vary significantly across primates. Here, we explore how these dental traits vary across mammals more broadly, focusing on terrestrial taxa in the clade of Boreoeutheria (Euarchontoglires and Laurasiatheria). We measured the postcanine teeth of *N* = 1,523 boreoeutherian mammals spanning six orders, 14 families, 36 genera, and 49 species to test hypotheses about associations between dental proportions and phylogenetic relatedness, diet, and life history in mammals. Boreoeutherian postcanine dental proportions sampled in this study carry conserved phylogenetic signal and are not associated with variation in diet. The incorporation of paleontological data provides further evidence that dental proportions may be slower to change than is dietary specialization. These results have implications for our understanding of dental variation and dietary adaptation in mammals.

## 1 INTRODUCTION

The evolution of the heterodont dentition in the late Triassic is widely appreciated as a key innovation contributing to the later evolutionary success of the mammalian class (Bergqvist, 2003; Butler, 2000; Cisneros, Abdala, Rubidge, Dentzien‐Dias, & Oliveira Bueno, 2011; Clemens, 1970, 1971; Hillson, 2005; Kermack & Kermack, 1984; Lucas, 2004; Lucas & Peters, 2000; Luo, Cifelli, & Kielan‐Jaworowska, 2001; McCollum & Sharpe, 2001; Muller & Wagner, 1991; Ungar, 2010; Zhao, Weiss, & Stock, 2000). The plesiomorphic mammalian dentition is characterized by four classes of teeth: incisors, canines, premolars, and molars, all of which can still be observed in most living mammals (Hillson, 2005). There is variation in the number, size, and shape of teeth across modern clades, with some mammals lacking entire tooth classes in both the maxilla and the mandible (e.g., the loss of canines and premolars in mice), and others having different numbers of maxillary and mandibular teeth and tooth class expression (e.g., Cetartiodactyla and Lepilemuridae; Line, 2003).

Since the very start of comparative anatomy, observed dental variation has provided insight into the broad range of foods that mammals consume (Cuvier, 1835). The number, size, and shape of teeth are strongly correlated with dietary specializations such as grazing, carnivory, insectivory, and gouging, among many others (Boyer, 2008; Boyer et al., 2010; Butler, 2000; Caumul & Polly, 2005; Hiiemae, 2000; Hunter & Jernvall, 1995). Morphological changes in adaptive dental phenotypes can often be tracked and associated with diet and ecology through evolutionary time, of which the most well‐cited example is hypsodonty in ungulates (Damuth & Janis, 2011; Strömberg, 2006; Williams & Kay, 2001). Additionally, changes in tooth proportions, for example, through carnassialization or reduction of the third molars, have also been linked to diet in some taxa (Carter & Worthington, 2016; Christiansen & Wroe, 2007). Consequently, dental features are frequently used in paleontology to reconstruct the diet of extinct mammals (Boyer, 2008; Boyer et al., 2010; Butler, 2000; Cardini & Elton, 2008; Caumul & Polly, 2005; Janis, 1984, 1997; Janis, Scott, & Jacobs, 1998; Jernvall, Hunter, & Fortelius, 1996; Ungar, 1998, 2017; Walker, 1981).

However, over the last 20 years, biologists have increasingly found evidence that the relationship between dental morphology and diet is not always clear‐cut. For example, stable isotopes and microwear have revealed changes in diet that are somewhat independent from changes in dental morphology (Bibi, Souron, Bocherens, Uno, & Boisserie, 2013; Feranec, 2003; Lister, 2013; MacFadden, Solounias, & Cerling, 1999; Sponheimer, Reed, & Lee‐Thorp, 1999). This apparent mismatch is likely driven by the observation that occlusal morphology can reflect adaptation to the most mechanically challenging foods a mammal processes independently of the frequency of that specific food in the diet (Ungar, 2009; Ungar, Healy, Karme, Teaford, & Fortelius, 2018), and that developmental mechanisms can evolve similarly but in response to different selective pressures, especially among closely related taxa (Ungar & Hlusko, 2016).

Dental phenotypes can also vary with life history traits like age at weaning, prenatal growth rates, and gestation length (Monson, Coleman, & Hlusko, 2019; Smith, 1989, 2018; Smith, Crummett, & Brandt, 1994). Other research has reported that some aspects of dental variation are more strongly associated with phylogenetic relatedness in mammals than diet or life history strategies (Gamarra, Delgado, Romero, Galbany, & Pérez‐Pérez, 2016; Macholán, 2006; Monson & Hlusko, 2018a, 2018b). These observations accord with results from comparisons between molecular and morphological data demonstrating that certain dental traits can reliably predict phylogenetic relatedness (Cardini & Elton, 2008; Caumul & Polly, 2005). These studies also suggest that the developmental etiology of dental variation may be a stronger evolutionary force than previously recognized. The canalization of development as a consequence of strong integration and genetic pleiotropy can act as a stabilizing selective pressure limiting rapid evolutionary change (Gibson & Wagner, 2000).

Due to recent advances in genotype:phenotype (G:P) mapping of mammalian dental variation, we can now approach the critical question that lies at the heart of comparative anatomy—to what degree is morphology evidence of a fine‐tuned response to selection versus a constrained result of stabilizing selection (Hlusko, 2004, 2016; Lovejoy, Cohn, & White, 1999)?

G:P mapping of dental patterning over the last two decades has led to a dramatic increase in our understanding of the genetic and developmental mechanisms that underlie mammalian dental variation (Bei, 2009; Hlusko, Sage, & Mahaney, 2011; Hlusko, Schmitt, Monson, Brasil, & Mahaney, 2016; Thesleff, 2006; Thesleff & Hurmerinta, 1981; Thesleff & Sharpe, 1997; Tucker & Sharpe, 2004). We now know that size variation in the anterior (incisor and canine) and posterior (premolar and molar) teeth is genetically independent in mammals, and as such, these represent two distinct genetic modules (Grieco, Rizk, & Hlusko, 2013; Hlusko et al., 2011). Within the postcanine module, premolars and molars represent two genetic modules that are influenced by different degrees of pleiotropy (Grieco et al., 2013; Hlusko et al., 2011). Within the molar module specifically, mouse development research has revealed that activating and inhibiting signals during development lead to the sequential and integrated development of the first through third molars, a process referred to as the inhibitory cascade (Kavanagh, Evans, & Jernvall, 2007). The inhibitory cascade model of molar size variation describes some mammalian clades better than others with support for this model published for some carnivorans and rodents (Evans & Jernvall, 2009), catarrhine primates (Schroer & Wood, 2015), and fossil mammals (Halliday & Goswami, 2013), whereas several other taxa are reported to deviate significantly from the predictions of the inhibitory cascade including South American ungulates (Wilson, Sánchez‐Villagra, Madden, & Kay, 2012), canids (Asahara, 2013), platyrrhine primates (Bernal, Gonzalez, & Perez, 2013), and voles (Renvoisé et al., 2009).

The vast majority of experimental developmental studies have occurred in the highly derived dentitions of mice (Thesleff, 2015, 2018). While this approach offers valuable insight into genetic mechanisms, it is important to keep in mind that this is evidence of the mechanisms that pattern the murine dentition. The distinct evolutionary history of rodents resulted in highly derived and reduced dentitions, a potentially significant caveat to the developmental genetics of this model system. Research that focuses on more evolutionarily conserved mammalian dentitions (primates, cervids, and equids) will provide essential insight into the more generalized genetic mechanisms that facilitated and constrained the evolution of mammalian dental variation and, consequently, this key mammalian innovation. To date, experimental manipulation of the development of mammalian dentitions with all four classes of teeth has been limited (but see Moustakas, Smith, & Hlusko, 2011). Much of our understanding of the G:P dental variation map for more generalized, evolutionarily conserved mammalian dentitions derives from quantitative genetic analysis of primates (Hlusko, 2004; Hlusko, Lease, & Mahaney, 2006; Hlusko & Mahaney, 2009; Rizk, Amugongo, Mahaney, & Hlusko, 2008).

The quantitative genetic approach to G:P mapping has revealed evidence of two independent genetic patterning mechanisms that influence dental proportions, or the relative sizes of teeth, in the postcanine dentition (Hlusko et al., 2016). Ratios of the mesiodistal dimensions of the fourth mandibular premolar:second mandibular molar (premolar–molar module, PMM) and first molar:third molar (molar module component, MMC) capture the phenotypic effects of these mechanisms (Hlusko et al., 2016). The PMM and MMC in primates are highly heritable, independent of body size, and underlain by as‐of‐yet uncertain genetic patterning mechanisms. The MMC is likely related to the inhibitory cascade. However, the inhibitory cascade is morphologically described by the two‐dimensional area for the first and third molars (mesiodistal length multiplied by buccolingual breadth; Kavanagh et al., 2007). Because earlier quantitative genetic research found that buccolingual breadth has pleiotropic effects with body size in primates (Hlusko et al., 2006), the MMC relies only on mesiodistal length. Therefore, and in light of the caveat we raised about the potentially derived developmental mechanisms of murines, we use a description of the anatomical structure to define this trait rather than referring to it by a presumed but unconfirmed developmental mechanism. Prior research shows that the MMC and PMM vary with strong taxonomic discrimination across extant and extinct primates (Hlusko et al., 2016). Here, we extend this research to test the hypothesis that PMM and MMC will have strong phylogenetic signals across mammals more broadly.

The permanent postcanine dentition (premolars and molars) develops and erupts throughout ontogeny in most boreoeutherian mammals with some species erupting their molars well after reproductive maturity (e.g., humans and suids; Hillson, 2005). Many life history traits emerge from coordinated changes during ontogeny (Stearns, 2000), and some aspects of dental variation have been shown to be associated with life history in mammals (e.g., timing of the eruption of the first molar, Smith et al., 1994) due in part to the slow development of the permanent dentition. However, recent work continues to emphasize the importance of considering phylogenetic relatedness when interpreting dental variation, as dental traits that were previously associated with life history have been shown to vary with conserved phylogenetic signal independent of life history when considered in a broader phylogenetic framework (Monson & Hlusko, 2018a, 2018b). As such, we considered both life history and phylogenetic relatedness in our investigation of postcanine dental proportions in mammals.

Here, we utilized a large morphological dataset spanning Boreoeutheria to assess how conserved or labile these two genetic patterning mechanisms (PMM and MMC) are in the evolution of mammalian dental variation. Boreoeutheria is comprised of two of the major extant eutherian mammalian clades that span a wide range of dietary, behavioral, and ecological adaptations and can be found on every major continent as well as in all major oceans: Euarchontoglires (primates and colugos, treeshrews, and rodents and lagomorphs) and Laurasiatheria (cetartiodactyl and perissodactyl ungulates [the former including cetaceans], carnivorans, pangolins, bats and flying foxes, and hedgehogs, shrews, moles, and solenodons; Nowak, 1999). Many species of nonboreoeutherian placental mammals, afrotherians and xenarthrans (e.g., sloths, anteaters, elephants, and armadillos), are characterized by highly derived and even absent dentitions (Hillson, 2005) and were not included in this study, although future studies that include these taxa may provide an illuminating comparison across mammals more broadly. Instead, for this study we focused exclusively on terrestrial boreoeutherian mammals with complete postcanine dentitions. Boreoeutherians are thought to have evolved approximately 100–80 Ma, with the first fossils definitively attributed to this clade dated to 65 Ma (Archibald, 2003; Kemp, 2005; O'Leary et al., 2013). This clade is ideal for our investigation as it encompasses the vast majority of extant eutherian mammals and a diverse array of dental variation and dietary niches. Additionally, as much of the work on mammalian dentition has focused on humans and other primates (Butler, 1963; Hlusko & Mahaney, 2009; Line, 2001; Townsend, Harris, Lesot, Clauss, & Brook, 2009), this study further contextualizes our understanding of the evolution of primate dental variation within boreoeutherian and mammalian evolution more broadly.

We assessed PMM and MMC across a large sample of mammals that includes *N* = 1,523 individuals spanning 14 families and two of the major eutherian clades: Euarchontoglires and Laurasiatheria (together known as Boreoeutheria). We combined this large dental phenotypic dataset with eight life history variables and adult body mass to test three hypotheses: (H1) Postcanine dental proportions (as captured by the MMC and PMM ratios) vary significantly across mammals; (H2) there is strong phylogenetic signal in postcanine dental proportions (MMC and PMM) across mammals; and (H3) variation in postcanine dental proportions (MMC and PMM) is associated with variation in diet and life history in mammals.

## 2 METHODS

### 2.1 Materials

The sample for this study includes *N* = 1,523 mammals spanning six orders, 14 families, 36 genera, and 49 species of Boreoeutheria (Table 1). We focused exclusively on terrestrial taxa with premolars and molars. These data represent the efforts of thousands of hours of data collection by more than a dozen researchers at 13 different museums across six countries (Table S1), and this is the most comprehensive investigation of mammalian dental proportions to date.

**Table 1:** Boreoeutherian species sampled in this study

Superorder	Order	Family	Species	Sample size (*n*)
Laurasiatheria	Carnivora	Canidae	*Canis latrans*	71
			*Urocyon cinereoargenteus*	35
			*Urocyon littoralis*	17
			*Vulpes vulpes*	10
		Ursidae	*Ursus americanus*	58
			*Ursus maritimus*	9
	Cetartiodactyla	Cervidae	*Blastocerus dichotomus*	6
			*Hippocamelus antisensis*	1
			*Hippocamelus bisulcus*	4
			*Mazama bricenii*	1
			*Muntiacus muntjak*	16
			*Odocoileus hemionus*	76
			*Ozotoceros bezoarticus*	4
			*Pudu mephistophiles*	2
			*Pudu puda*	4
			*Rangifer tarandus*	8
		Hippopotamidae	*Choeropsis liberiensis*	22
			*Hippopotamus amphibius*	114
		Suidae	*Hylochoerus meinertzhageni*	40
			*Potamochoerus larvatus*	71
			*Potamochoerus porcus*	41
			*Potamochoerus* sp.	6
	Chiroptera	Pteropodidae	*Dobsonia minor*	14
			*Dobsonia moluccensis*	5
			*Pteropus conspicillatus*	20
			*Pteropus mariannus*	30
			*Pteropus woodfordi*	2
			*Rousettus amplexicaudatus*	31
	Perissodactyla	Equidae	*Equus burchelli*	7
			*Equus caballus* (*ferus*)	5
			Total	730
Euarchontoglires	Primates	Atelidae	*Alouatta palliata*	28
		Cercopithecidae	*Cercocebus atys*	4
			*Cercocebus galeritus*	1
			*Cercocebus torquatus*	10
			*Cercopithecus mitis*	81
			*Chlorocebus aethiops*	8
			*Colobus guereza*	112
			*Macaca fascicularis*	74
			*Macaca mulatta*	67
			*Nasalis larvatus*	25
			*Papio hamadryas*	56
			*Presbytis melalophos*	76
			*Presbytis rubicunda*	74
			*Theropithecus gelada*	7
		Gorillidae	*Gorilla gorilla*	41
		Hominidae	*Homo sapiens*	25
		Panidae	*Pan paniscus*	30
			*Pan troglodytes*	54
		Pongidae	*Pongo pygmaeus*	8
	Rodentia	Chinchillidae	*Lagostomus maximus*	12
			Total	793
			TOTAL	1,523

### 2.2 Data collection and analytical methods

We assessed only adult individuals with complete postcanine dentitions (fourth premolars [P4] to third molars [M3]). As MMC and PMM were described for the mandibular dentition of primates (Hlusko et al., 2016), and because many laurasiatherian mammals have third molars in the mandible and not the maxilla, we focused on mandibular dentitions for this study.

We took the length of the mandibular premolars and molars of each individual using Mitutoyo calipers according to previously described protocols (Grieco et al., 2013). Length was measured as the mesiodistal length with some variation based on positioning of the teeth. Due to the immensity of the data collection undertaken, multiple researchers took measurements, specializing on subclades of the larger sample. While not all measurements were taken by a single researcher, data collection of dental linear metrics has been common practice for over a century, and all researchers followed standardized protocols (Grieco et al., 2013). In cases where multiple researchers collected measurements for the same taxa, interobserver error was calculated by taking the average difference between each pair of measurements and dividing by the sample mean for that metric to calculate measurement error as a percentage of the mean for the taxon. Measurements were only included if error was under 5%. Information on which researchers took measurements for each taxon is available as part of Table S1. For taxa measured by multiple researchers, the mean for each specimen is reported.

Taxa included in this study are held at the following museums: American Museum of Natural History, New York, New York, USA; Cleveland Museum of Natural History, Cleveland, Ohio, USA; Forschungsinstitut und Naturmuseum Senkenberg, Frankfurt, Germany; Musée des Confluences, Lyon, France; Musée Royal de l'Afrique Centrale, Tervuren, Belgium; Muséum d'Histoire Naturelle, Berne, Switzerland; Muséum d'Histoire Naturelle de la Ville de Genève, Switzerland; Muséum National d'Histoire Naturelle, Paris, France; Museum für Naturkunde, Berlin, Germany; Museum of Vertebrate Zoology, Berkeley, California, USA; Natural History Museum, London, UK; Phoebe A. Hearst Museum, Berkeley, California, USA; and Smithsonian National Museum of Natural History, Washington, D.C., USA.

We used the left side of the dentition unless measurements could not be taken, in which case we used the right side of the dentition in the analyses. For taxa that were measured by multiple investigators (e.g., *Lagostomus*; see Table S1 for more details), a subsample of ten specimens was measured three times to confirm that interobserver error was <5%. Although there is no evidence that MMC and PMM vary between sexes (Hlusko et al., 2016; data herein), in all cases in this study, effort was made to have balanced samples of males and females. The MMC and PMM values of each individual included in this study are available in the Supporting Information. There is particularly good coverage of primates, but further taxonomic coverage in future studies, particularly of Rodentia, will provide increased resolution and likely strengthen the results of the study, as unbalanced sampling may affect phylogenetic analyses.

All statistical analyses were conducted in R version 3.2.3 (R Core Team, 2016). First, we calculated the MMC (mesiodistal length of M_3_ divided by mesiodistal length of M_1_) and PMM (mesiodistal length of M_2_ divided by mesiodistal length of P_4_) ratios of dental length according to previous protocols (Hlusko et al., 2016). As the ratios are unit‐free and calculated consistently across all taxa, and as a previous quantitative genetics study found no correlation between body size and these ratios (Hlusko et al., 2016), no other size correction was done for these two traits. Next, we conducted a series of descriptive statistics by order, family, and genus using the describe By function in *psych* (Revelle, 2015).

We produced bivariate plots comparing MMC and PMM across Boreoeutheria at several taxonomic levels (genus, family, order) using qplot in *ggplot2* (Wickham, 2016). To compare MMC and PMM across families (H1), we conducted a phylogenetic ANOVA using the aov.phylo function in *geiger* and a published mammalian phylogeny (Faurby & Svenning, 2015; Harmon et al., 2016). We trimmed the phylogeny according to the species included in our sample. All species in our sample were represented in the phylogeny except for *Equus burchelli*, *Mazama bricenii*, and *Odocoileus hemionus*, and as such these taxa were excluded from the phylogenetic analyses and included only in the descriptive statistics and bivariate plots.

In order to test for phylogenetic signal in the dental ratios and all life history variables (H2), we conducted tests for Blomberg's *K* and Pagel's lambda (Kamilar & Cooper, 2013). For Blomberg's *K*, a value >1 suggests a stronger phylogenetic signal than expected under Brownian motion (BM), while a value equal to 1 suggests that the traits vary along the phylogeny in a manner consistent with BM, and a value <1 suggests that the traits vary along the phylogeny in a manner that is more random than expected under BM and may be the result of selection on those phenotypes (Blomberg, Garland, & Ives, 2003). For Pagel's lambda, a value closer to 1 indicates higher phylogenetic signal, while a value closer to 0 indicates lower phylogenetic signal. Both analyses test for phylogenetic signal but under different frameworks (Blomberg et al., 2003; Pagel, 1999). For Blomberg's *K*, the variance is partitioned according to clades, where a *K* > 1 indicates significant variance between clades, and a *K* < 1 indicates variance within clades (Blomberg et al., 2003; Molina‐Venegas & Rodríguez, 2017). In contrast, Pagel's lambda tests for similarity of covariance among species against covariance expected under a BM model (Molina‐Venegas & Rodríguez, 2017; Pagel, 1999). Variation in the availability of life history data results in different species sample sizes for each trait. Additionally, more comprehensive taxonomic sampling across clades will likely improve our understanding of the relationship between postcanine dental variation and phylogenetic relatedness in mammals.

To further interpret phylogenetic signal and better contextualize the evolution of dental proportions (H2), we estimated ancestral mammalian MMC and PMM values, as well as ancestral values for the life history variables, and generated a series of ancestral state reconstructions (ASR) using contMap in *phytools* (Revell, 2012) which maps continuous variables along a phylogeny of interest. We quantified the estimated values at internal nodes using fastAnc in *phytools* (Revell, 2012), a function that generates maximum‐likelihood ancestral states for continuous traits. Because there is some evidence that ratios can be poorly modeled by Brownian motion (ratios are unlikely to increase linearly with time), we also ran an ancestral state reconstruction using dental lengths and calculated the ancestral MMC and PMM using reconstructed ancestral dental lengths.

To investigate potential correlates with MMC and PMM variation, we collected data on life history variables from the AnAge database, part of the Human Ageing Genomic Resources database (Tacutu et al., 2013). In each case, we used the species average of MMC and PMM. Previous studies have linked life history variables such as longevity and age at weaning to the timing of tooth formation in primates (Smith, 1989; Smith et al., 1994), and recent work hypothesized that variation in prenatal growth rates is associated with tooth number and development of the postcanine dentition (Monson et al., 2019). Building from this literature, we compared dental metrics and proportions with a series of life history variables in this study including gestation length (days), birth weight (grams), adult weight (grams), longevity (years), litter size, age at weaning (days), and age at female sexual maturity (days). We also calculated prenatal and postnatal growth rates according to standard protocols, where prenatal growth rate is the ratio of birth weight to gestation length, and postnatal growth rate is the ratio of adult weight to days to sexual maturity (Montgomery & Mundy, 2013). All life history and body size variables were log‐transformed for analyses with the exception of litter size. It has been previously hypothesized that slower prenatal growth rates can lead to reduction or complete lack of development of the third molars in primates (Monson et al., 2019). The first and second generations of mammalian teeth begin developing *in utero* and finish erupting well past sexual maturity in many taxa (e.g., humans) and are thereby subject to many stages of life history including gestation, labor and delivery, weaning, and sexual maturity (Smith, 2018; Tucker & Sharpe, 2004). Based on previous work on primate dental proportions (Hlusko et al., 2016; Monson et al., 2019), this study predicts a correlation between MMC and/or PMM and life history in boreoeutherian mammals (H3).

In order to assess any correlations between MMC and PMM and diet in our sample, we collected data on diet from the literature (Nowak, 1999). Animals were classified into one of six dietary categories based on their dominant food sources as detailed by a compilation of observational, fecal, and stomach content studies in Nowak (1999): carnivore, folivore, frugivore, granivore, grazer, and omnivore. The original sources referenced in this compilation of studies varied in method used to determine preferred food type, an important caveat when considering relationships between diet and morphology. Information on classification of individual species can be found in the life history and diet dataset, available in Table S2.

To directly compare life history variables with variation in MMC and PMM (H3), we ran a series of phylogenetic independent contrasts using the crunch function in *caper* (Orme et al., 2013). The crunch algorithm calculates linear models comparing continuous traits, here MMC and PMM, and the life history variables of interest. Additionally, we generated a bivariate plot comparing MMC and PMM across dietary categories in Boreoeutheria.

Because diet as defined here is a discrete, categorical variable, we compared variation in MMC and PMM with diet using phylogenetic generalized least squares (PGLS) analyses in *caper* (Orme et al., 2013). Phylogenetic generalized linear analyses fit models between the traits of interest (here the MMC and PMM ratios, and dietary category) taking into account phylogenetic nonindependence and outputting a coefficient of determination and significance for the sample as a whole as well as comparing interactions between dependent variables (here, diet; Orme et al., 2013). Because the PGLS analysis chooses a reference variable to which it compares the other dependent variables based on alphabetical order, and because we have unequal sample sizes in our dietary categories, we ran two PGLS analyses: one where the reference category is carnivore (the smallest representative sample), and one where the reference category is grazer. It is important to note that transitions between dietary categories are not equally easy, and there is evidence that acquiring and processing food can have scaling effects that result in correlations between body size and diet (e.g., Kay's threshold; Gingerich, 1980; Jones, Rose, & Perry, 2014).

As tooth length is a long‐standing metric for investigating diet and body size, and dental proportions are calculated from tooth lengths, we regressed individual tooth lengths against cube root body mass and compared the residuals for each tooth against diet across the phylogeny in a PGLS analysis to further compare variation in these traits. Cube root body mass was used here to account for scaling and allometric relationships between tooth length and adult body mass (Ungar, 2015).

## 3 RESULTS

Our results demonstrate that suid genera *Hylochoerus* and *Potamochoerus* have the highest MMC values, and *Hylochoerus* and the ursid genus *Ursus* have the highest PMM values of the taxa sampled, likely driven by the elongate third and second molars in these taxa. In contrast, other genera in Carnivora and Chiroptera have the lowest MMC and PMM values of all sampled taxa (Table 2, Table S3), likely driven by the reduced third and second molars in these taxa. Comparisons using phylogenetic ANOVA show that dental proportions vary significantly across families of Boreoeutheria for both MMC (*R* ^2^ = 0.9012, *p* < 0.0001) and PMM (*R* ^2^ = 0.7422, *p* < 0.0001). This significance is driven by Canidae (*p* < 0.0001), Hippopotamidae (*p* = 0.036), Pteropodidae (*p* = 0.001), and Suidae (*p* < 0.0001) for MMC, and Canidae (*p* = 0.0002), Chinchillidae (*p* = 0.01), Equidae (*p* = 0.01), and Pteropodidae (*p* < 0.0001) for PMM. Descriptive statistics by genus and family are presented in Tables S3 and S4.

**Table 2:** Summary statistics for MMC and PMM by order

Order	Trait	Sample size (*n*)	Mean	*SD*	Range	Skew	Kurtosis	*SE*
Carnivora	MMC	200	0.43	0.27	0.91	0.74	−1.30	0.02
	PMM	200	1.21	0.54	1.64	0.72	−1.29	0.04
Cetartiodactyla	MMC	416	1.88	0.56	2.90	0.98	0.11	0.03
	PMM	416	1.46	0.25	1.70	1.54	3.42	0.01
Chiroptera	MMC	102	0.46	0.11	0.40	0.43	−1.04	0.01
	PMM	102	0.81	0.05	0.24	−0.57	0.18	0.00
Perissodactyla	MMC	12	1.27	0.21	0.72	−0.80	−0.20	0.06
	PMM	12	0.96	0.05	0.17	0.85	−0.07	0.01
Primates	MMC	781	1.23	0.20	1.10	0.37	−0.71	0.01
	PMM	781	1.29	0.20	0.93	−0.01	−1.23	0.01
Rodentia	MMC	12	0.87	0.05	0.17	−0.34	−0.97	0.01
	PMM	12	0.96	0.09	0.35	0.48	−0.24	0.03

Abbreviations: MMC: molar module component; *n*: sample size; PMM: premolar–molar module; *SD*: standard deviation; *SE*: standard error.

**Figure 1:**
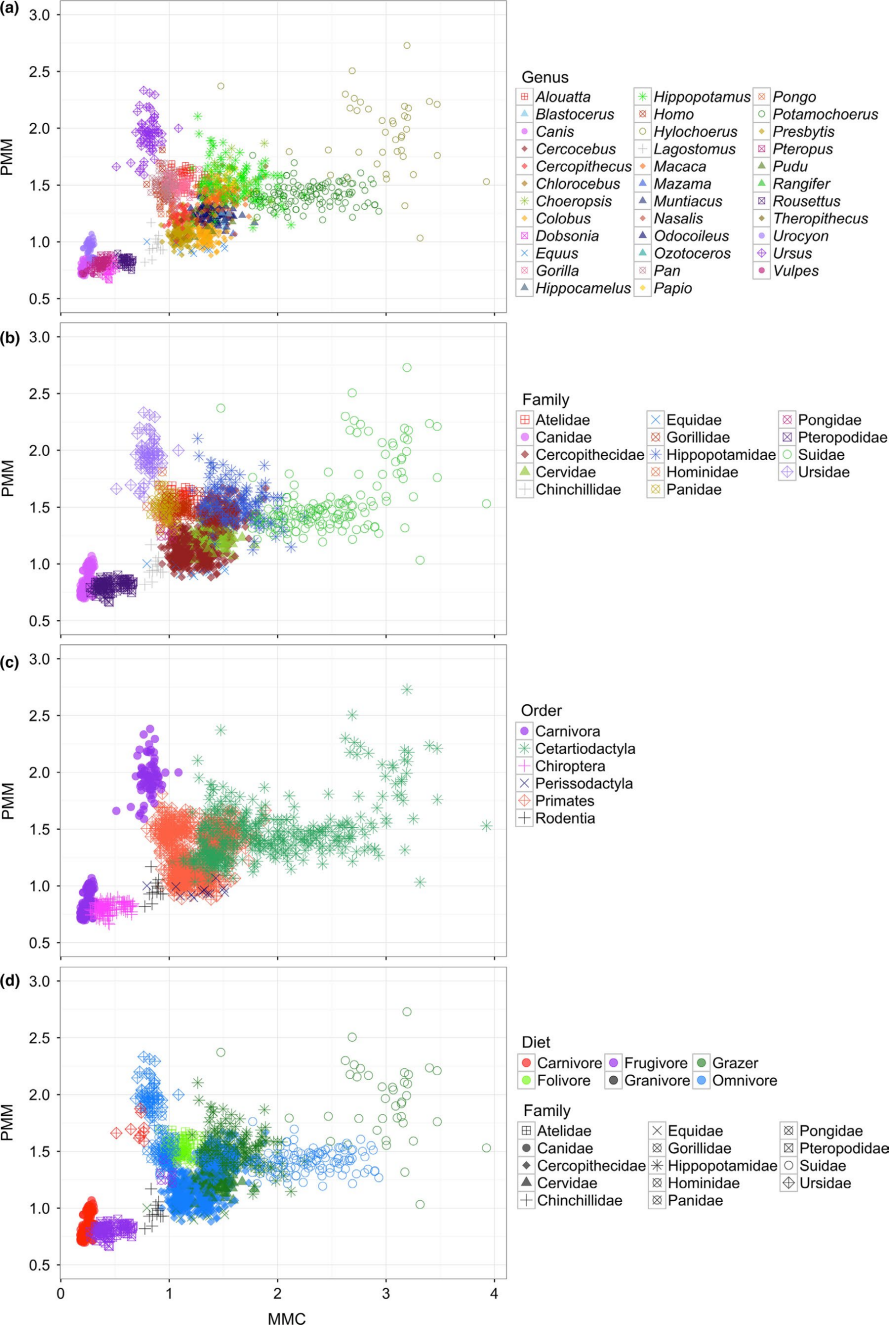
Variation in MMC and PMM. See figure for legends. (a) Genus‐level variation, (b) family‐level variation, (c) order‐level variation, and (d) variation coded by diet

Visualization of MMC and PMM in bivariate space demonstrates clear taxonomic discrimination using these traits at both the family and genus levels and provides further support for the strong phylogenetic signal observed here (Figure 1a,b). In contrast, there is very little taxonomic discrimination when considering MMC and PMM at the level of order, driven largely by the separation between Ursidae and Canidae and the wide dispersion of values for Cetartiodactyla. There is also no clear pattern of discrimination by diet, reinforcing the lack of significant association between these traits in a phylogenetic context in this sample (Figure 1c,d).

Using Pagel's lambda, MMC and PMM, as well as all life history variables considered here, have significant phylogenetic signals approaching 1. There are some differences in phylogenetic signal using Blomberg's *K*. Molar module component has the highest *K*‐value and significant phylogenetic signal (*p* = 0.001). Postnatal and prenatal growth rates, and age at sexual maturity, also have *K*‐values ≥1 and significant phylogenetic signals. All other life history traits and PMM have *K*‐values <1 indicating a significant deviation from Brownian motion and suggesting that selective pressures may be impacting the distribution of these phenotypes across the phylogeny (Table 3).

**Table 3:** Results of the tests for phylogenetic signal

Trait	Sample size (*n*)	Blomberg's *K*	*K* (*p*)	Lambda
MMC	46	1.355	**0.001**	0.999
Postnatal growth rate	31	1.068	**0.037**	1.000
Sexual maturity (F, days)	31	1.004	**0.002**	0.971
Prenatal growth rate	32	1.000	**0.017**	1.000
Gestation (days)	37	0.898	**0.001**	0.962
Litter size	38	0.871	**0.001**	1.000
Longevity (yrs)	30	0.776	**0.001**	0.985
Weaning (days)	31	0.771	**0.001**	0.928
Birth weight (g)	32	0.571	**0.001**	0.970
Adult weight (g)	38	0.485	**0.001**	0.965
PMM	46	0.427	**0.001**	0.965

All measurements were log‐transformed prior to analysis except for MMC, PMM, and litter size. Abbreviations: F: female; g: grams; *K*: Blomberg's *K*; MMC: molar module component; *n*: sample size; *p*: *p*‐value; PMM: premolar–molar module; yrs: years. All *K p*‐values are significant (*p* < 0.05 in bold). Sample size is number of species.

**Figure 2:**
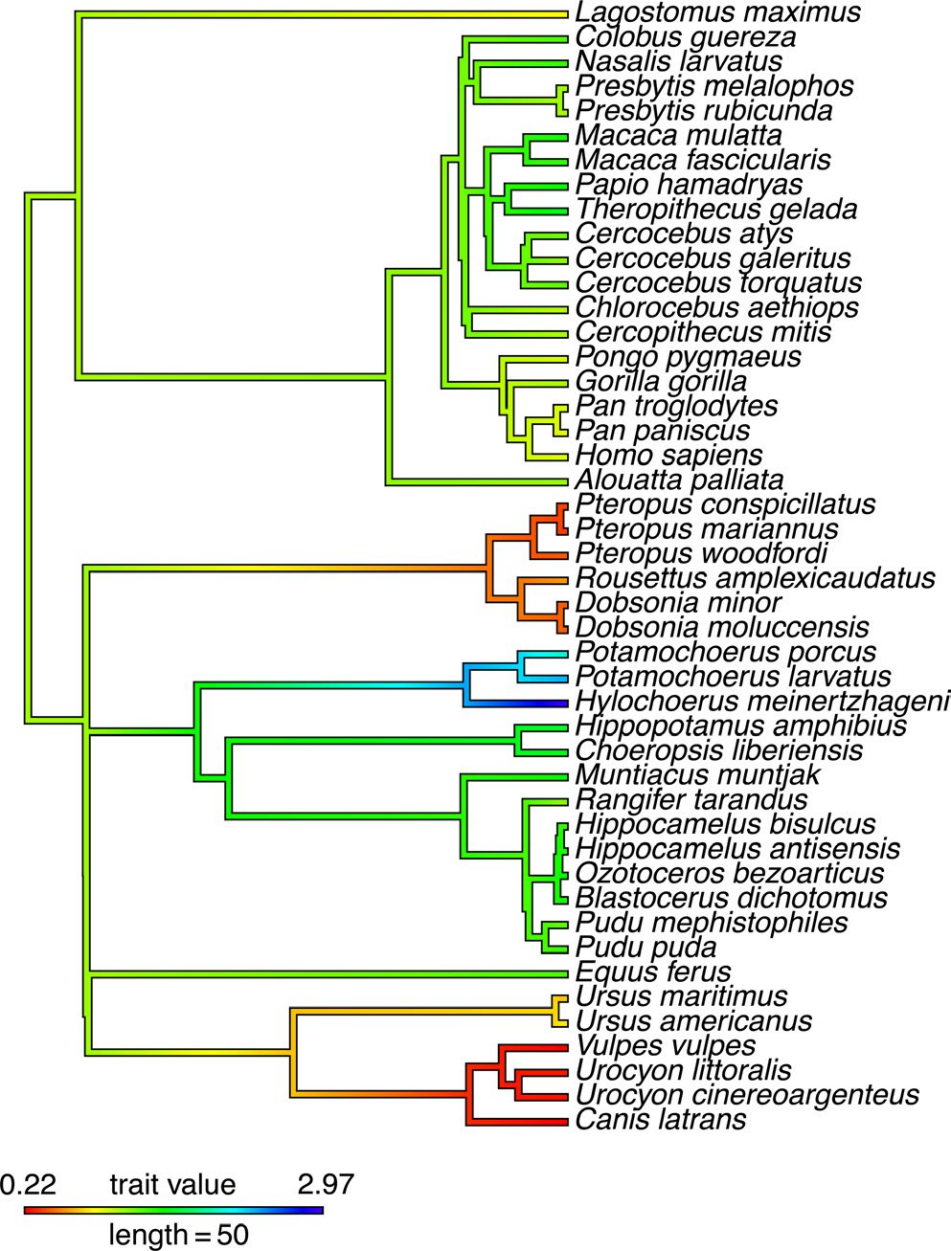
Ancestral state reconstruction of MMC values in Boreoeutheria. See Table S5 and Figure S1 for supported MMC values at each ancestral node

Ancestral state reconstruction tracks changes in MMC and PMM across the boreoeutherian phylogeny and provides support for derived MMC values in Pteropodidae, Canidae, and Suidae, with notable although lesser changes in Ursidae and Chinchillidae (Figure 2). In contrast, other families in Primates, Cetartiodactyla, and Perissodactyla retain more ancestral MMC values comparable to the ancestors of Laurasiatheria and Euarchontoglires which are supported to have MMC values of 1.13 and 1.10 respectively (Table S5, Figure S1). Of the extant clades sampled here, the ancestor of all primates, the ancestor of anthropoid primates, and the ancestor of *Presbytis* are supported to have MMC values most similar to the MMC values reconstructed for the common ancestor of Euarchontoglires and Boreoeutheria more generally (Table S5, Figure S1).

**Figure 3:**
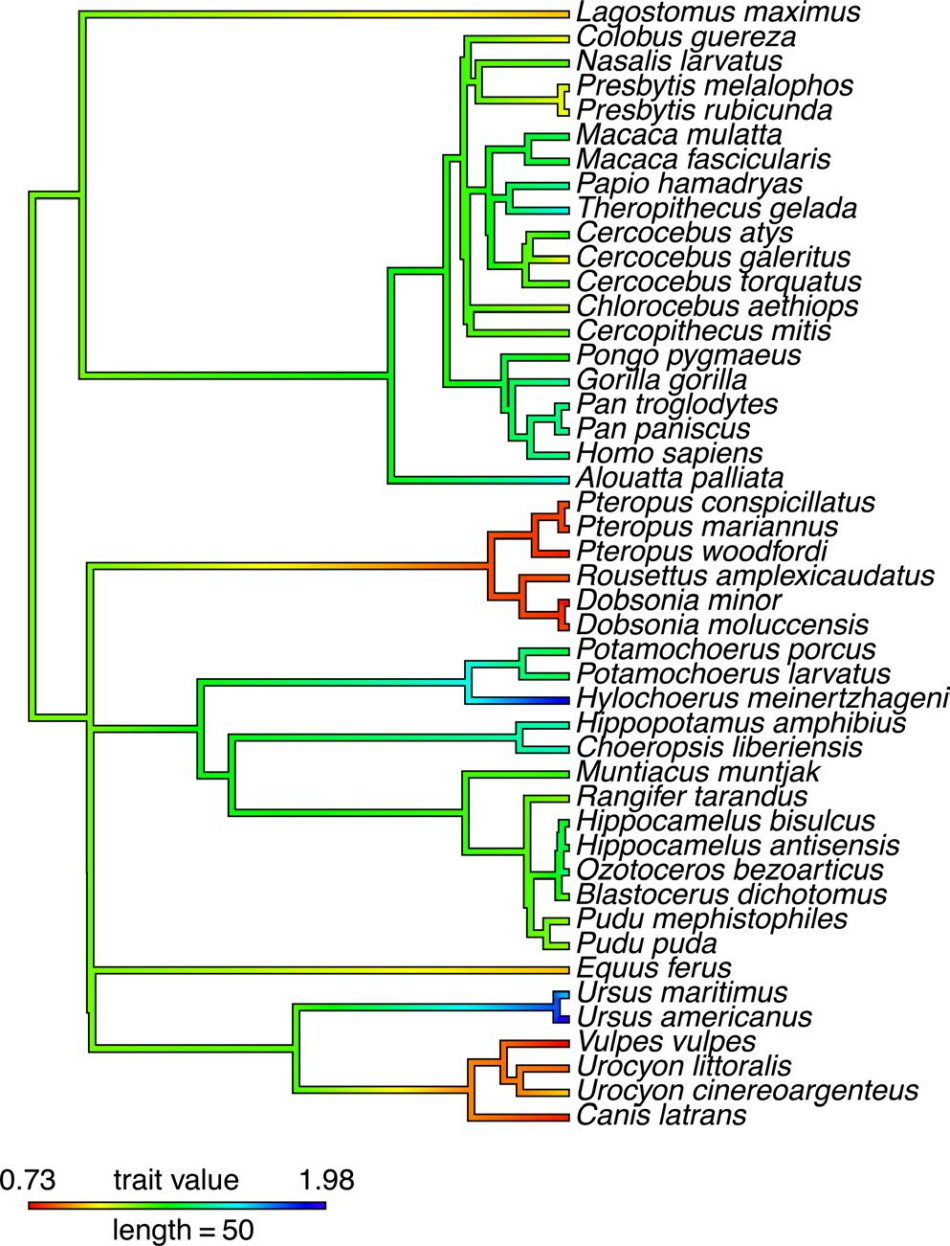
Ancestral state reconstruction of PMM values in Boreoeutheria. See Table S5 and Figure S2 for supported PMM values at each ancestral node

Premolar–molar module has a similar distribution of extant and ancestral values with ancestral state reconstruction supporting derived PMM values in Pteropodidae, Canidae, Ursidae, and the suid *Hylochoerus*, and ancestral PMM values of approximately 1.2 for the ancestors of Laurasiatheria and Euarchontoglires (Figure 3). Like with the MMC values, almost all primates and cetartiodactyls retain more ancestral PMM values. The primate ancestor is supported to have a PMM value of 1.35, and the ancestor of Euarchontoglires is supported to have a PMM value of 1.2 like the ancestor of Boreoeutheria more generally (Table S5, Figure S2). Interestingly, due to the divergence in PMM values between Ursidae and Canidae, the ancestor of Carnivora is also supported to have a PMM value similar to the ancestor of Laurasiatheria and Boreoeutheria (1.25, 1.20, and 1.20, respectively). Within primates, *Cercocebus* has a PMM value most similar to the ancestral predictions (1.24; Table S5, Figure S2). Overall, extant African and Asian monkeys (Cercopithecidae) have dental proportions most similar to the ancestral MMC and PMM values predicted by ancestral state reconstruction in this study. This is supported both when ASR is applied to the MMC and PMM ratios and when ASR is applied to raw dental lengths and MMC and PMM are calculated from reconstructed ancestral values (Table S6, Figure S3).

The coefficients of determination comparing life history traits with MMC and PMM are not significant, indicating that there is no consistent relationship between these variables in a phylogenetic context. Variation in life history traits is not associated with variation in MMC and PMM values (Table 4). There is also no significant relationship between dietary category and MMC or PMM in a phylogenetic context (MMC: *p* = 0.1381, *R* ^2^ = 0.0795; PMM: *p* = 0.07569, *R* ^2^ = 0.1165). While grazers and carnivores have MMC and PMM values that are significantly different from each other, PGLS analyses find no significant association between dietary category and MMC and PMM variation when phylogeny is taken into account. However, when regressing individual tooth lengths against cube root body mass and comparing the residuals for each species against diet in PGLS, we find that the residuals are significantly associated with diet for all teeth (*p* < 0.001) with greatest significance in the first molar (Table 5).

**Table 4:** Phylogenetic independent contrasts comparing life history variables and MMC and PMM across boreoeutherian mammals

Trait	Sample size (*n*)	MMC (*R* ^2^)	MMC (*p*)	PMM (*R* ^2^)	PMM (*p*)
Sexual maturity (F, days)	31	0.087	0.059	−0.013	0.436
Litter size	38	0.046	0.104	0.027	0.162
Gestation (days)	37	0.014	0.228	−0.024	0.686
Postnatal growth rate	31	0.013	0.246	0.026	0.190
Adult weight (g)	38	−0.012	0.463	0.002	0.307
Prenatal growth rate	32	−0.025	0.615	−0.017	0.499
Birth weight (g)	32	−0.026	0.638	0.030	0.173
Weaning (days)	31	−0.033	0.818	−0.031	0.748
Longevity (yrs)	30	−0.036	0.992	−0.035	0.908

Abbreviations: F: female; g: grams; MMC: molar module component; *n*: sample size; *p*: *p*‐value; PMM: premolar–molar module; *R* ^2^: coefficient of determination; yrs: years. Sample size is number of species. Note that none of the phylogenetic independent contrasts are significant (*p* > 0.05).

**Table 5:** Results of the PGLS analysis comparing tooth length, body size, and diet

Tooth	Metric	Cube root body mass (*R* ^2^)	Cube root body mass (*p*)	Diet (*R* ^2^)	Diet (*p*)
DP4L	Raw	0.7636	**0.0000**	–	–
	PGLS residual	–	–	0.4853	**0.0001**
DM1L	Raw	0.8265	**0.0000**	–	–
	PGLS residual	–	–	0.7271	**0.0000**
DM2L	Raw	0.9326	**0.0000**	–	–
	PGLS residual	–	–	0.3964	**0.0006**
DM3L	Raw	0.7887	**0.0000**	–	–
	PGLS residual	–	–	0.2726	**0.0085**

Abbreviations: D: mandibular; L: length; M: molar; P: premolar; *p*: *p*‐value; PGLS: phylogenetic generalized least squares; *R* ^2^: coefficient of determination. DM1L is mandibular first molar length. All PGLS regressions are highly significant (*p* < 0.01 in bold).

## 4 DISCUSSION

Tooth size, dental proportions, and tooth crown morphology have all been used as proxies for the interpretation of diet in the fossil record (Boyer, 2008; Cardini & Elton, 2008; Caumul & Polly, 2005; Fortelius, Made, & Bernor, 1996; Janis, 1984, 1997; Janis et al., 1998; Jernvall et al., 1996; Ungar, 1998, 2017; Walker, 1981). We analyzed ratios (PMM and MMC) that reflect the phenotypic output of two genetic patterning mechanisms on the mammalian postcanine module. Our data demonstrate that the relative sizes of premolar and molar teeth, as captured by the MMC and PMM ratios of dental length, are significantly different across boreoeutherian mammals and have strong phylogenetic signal. We interpret this association with phylogenetic relatedness to be evidence that tooth proportionality is highly conserved over evolutionary time, and variation in dental proportions, particularly molar proportions, generally reflects variation in phylogeny over variation in diet. This is shown through tests of phylogenetic signal as well as clear taxonomic discrimination at the genus and family levels in bivariate space. In contrast, the MMC and PMM traits do not vary significantly with diet in a phylogenetic context at this broad taxonomic scale. Some previous studies have associated variable proportions of postcanine tooth length to diet in primates (Asahara, 2013; Lucas, Corlett, & Luke, 1986), but our more taxonomically comprehensive study reveals significant phylogenetic signal that is largely independent of variation in diet, although some individual taxa have taxon‐specific dental adaptations that contribute to variation in the MMC and PMM (Asahara & Takai, 2017). This suggests that MMC and PMM may evolve in tandem with the morphology of taxon‐specific dental adaptations, such as the carnassials of carnivorans, and the reduced third molars of carnivorans and bats.

While variation in dietary strategies within clades is statistically independent of changes in relative postcanine dental proportions (suggesting that dental proportions contribute less to dietary adaptations than do other cranial and dental phenotypes), individual tooth length measurements relative to adult body mass are significantly correlated with diet. This is consistent with previous studies that found significant allometric relationships between tooth length and body mass, and significant associations between diet and individual tooth lengths (Asahara & Takai, 2017; Copes & Schwartz, 2010; Scott, 2011; Scott et al., 2018). This significant relationship is also likely influenced by ancestral dietary “bauplans” of different clades, where individual teeth have evolved unique morphologies as functional adaptations to processing particular foods (Hunter & Jernvall, 1995; Kay, 1975, 1977; Lucas, 1980; Ungar, 2009; Ungar et al., 2018). The length of the mandibular first molar is most significantly correlated with diet in our sample, likely related to the modification of this tooth into a carnassial for the processing of animal tissues in many species of Carnivora (Asahara & Takai, 2017).

Adaptations to increased biomechanical torque and lever forces associated with the enlarged P^4^/M_1_ carnassial complex (Van Valkenburgh, 1991) also likely contribute to the dietary trend identified in our data where all carnivorous species sampled have an average MMC that is <1. Omnivorous members of Carnivora, represented here by *Ursus americanus*, also have an MMC <1, likely retained from their ancestral dental “bauplan” which included carnassials (Butler, 1946). Less is known about the dental proportions of carnivorous mammals in other orders such as the Tasmanian devil (*Sarcophilus harrisii*), a marsupial that does not technically have carnassials but does have long shearing blades and retains four molars in the adult dentition (Marshall & Corruccini, 1978; de Muizon & Lange‐Badré, 1997; Werdelin, 1987, 1988).

One clear example of the disjoint between proportions of dental length and diet is the polar bear (*Ursus maritimus*), a carnivorous species that evolved relatively recently, over the last 700,000 years (Cahill et al., 2013; Edwards et al., 2011; Hailer et al., 2012; Kurtén, 1964; Slater, Figueirido, Louis, Yang, & Valkenburgh, 2010; Talbot & Shields, 1996). While polar bears exhibit reduced surface area of the postcanine dentition, a feature associated with increased carnivory (Slater et al., 2010), the relative proportions of their postcanine teeth are much more similar to those of their omnivorous relatives and distinct from other carnivorous mammals (e.g., Canidae). *t* Tests conducted in R (R Core Team, 2016) comparing MMC and PMM indicate significant differences between Ursidae and Canidae (*p* < 0.0001), while the MMC ratios of Ursidae and Chinchillidae (Tukey's HSD, *p* = 0.9997), Ursidae and Pongidae (Tukey's HSD, *p* = 0.4987), and Ursidae and Hominidae (Tukey's HSD, *p* = 0.073), all omnivorous and granivorous animals, do not differ significantly. Likewise, *t* tests comparing polar bears (*Ursus maritimus*) with Canidae indicate significant differences between these taxa (MMC: *p* < 0.0001; PMM: *p* < 0.0001). This example provides some insight into the pace of evolution of dental proportions. Despite the intense carnivory of polar bears over the last 700,000 years, their MMC and PMM values have not deviated significantly from their omnivorous phylogenetic roots. A deeper investigation of the evolution of PMM and MMC in Ursidae, and especially the folivorous giant panda (*Ailuropoda melanoleuca*), would offer further insight into dental evolution in this family. Additionally, assessing MMC and PMM in a clade with several taxa with highly divergent/specialized diets could give us a better idea of the extent of phylogenetic inertia in these traits and further refine the timeline for significant morphological divergence in dental proportions.

We also found a lack of correlation between postcanine dental proportions and life history characteristics. Some aspects of the dentition, such as rates and timing of enamel deposition, provide essential insight into variation in life history (Smith, 2018). Our results demonstrate that other aspects of the dentition are decoupled from life history as has been seen in other studies (Monson & Hlusko, 2018a, 2018b). Our analyses indicate that life history and diet may be more evolutionarily labile than postcanine dental proportions and, as such, more responsive to selective pressure. In contrast, postcanine dental proportions likely require significant selective pressure over long timescales to diverge from the ancestral condition. Several mammalian lineages are characterized by significant deviation from early mammals suggesting that they experienced bouts of strong evolutionary pressure (e.g., murines).

To better understand the evolution of postcanine dental proportions in mammals, we performed a subsample analysis comparing our ancestral state reconstructions with data from the fossil record, collecting data on seven fossil species from six genera representing three fossil groups spanning Oligocene to Pleistocene: fossil Ursidae (*Arctotherium brasiliense*, Trajano & Ferrarezzi, 1994; *Cyo* *narctos dessei*, de Bonis, 2013; *Ursavus tedfordi*, Qiu, Deng, & Wang, 2014), Amphicynodontinae (*Campylocynodon personi*, Chaffee, 1954), and archaic ungulates (*Oxyacodon agapetillus* and *O. priscilla*, and *Protungulatum mckeeveri*, Archibald, 1982). Of the fossils sampled, the archaic ungulates are the most ancient, dated to the early Paleocene of North America (Archibald, 1982; Archibald, Schoch, & Rigby, 1983). Of the carnivorans, *Arctotherium* is the most recent, dated to the Pleistocene (Trajano & Ferrarezzi, 1994). *Cyonarctos* and *Ursavus* are dated to the Oligocene and Miocene, respectively (de Bonis, 2013; Qiu et al., 2014), and *Campylocynodon* (alternately classified as *Parictis*; Clark & Guensburg, 1972) is dated to the Oligocene of Europe and North America (Chaffee, 1954).

**Figure 4:**
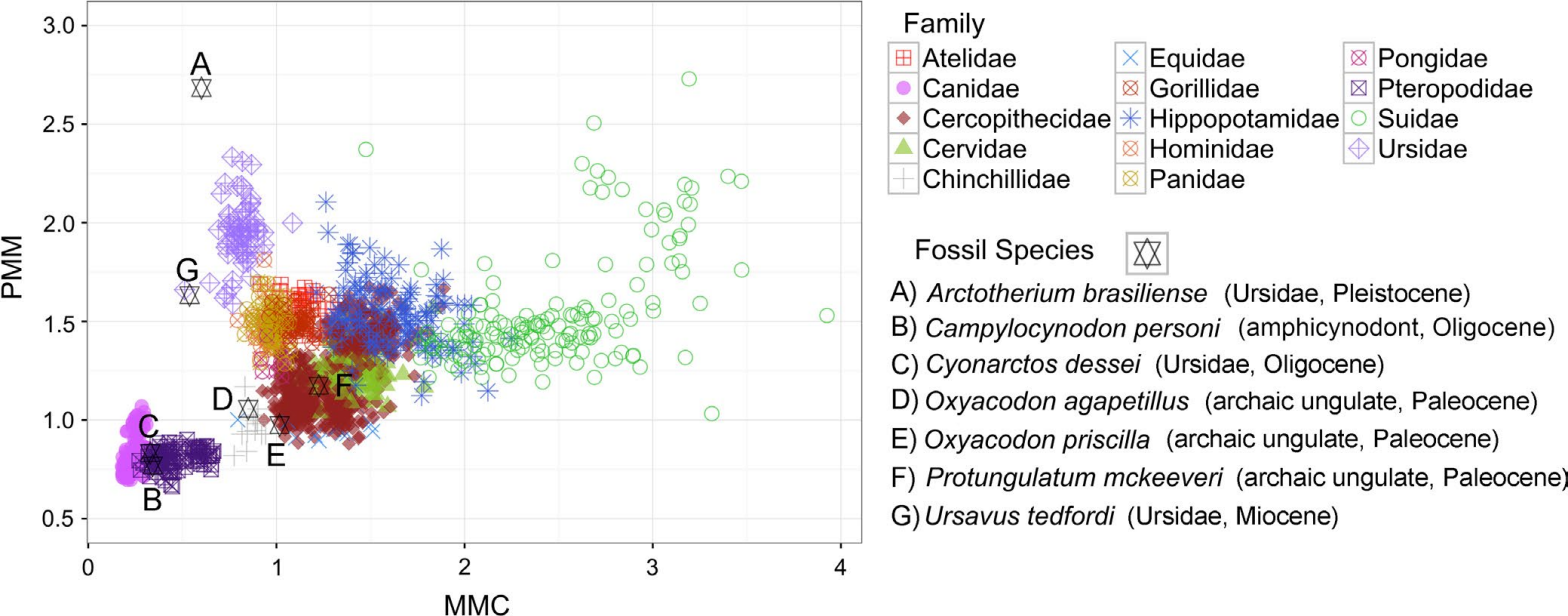
Variation in MMC and PMM visualized at family‐level with fossil species marked with a star and indicated by an uppercase letter. Broader taxonomic affiliation and geological ages of fossils are in parentheses following the species name. See figure for legend

The inclusion of fossil data into our plots of MMC and PMM demonstrates that the oldest fossils (archaic ungulates) fall close to the 1:1 axis of MMC and PMM variation (Figure 4) near the predicted ancestral condition for Boreoeutheria (1.1, 1.2; Table S5). This pattern provides further evidence that ancestral mammals had more homogeneous postcanine dental proportions as has been noted in previous studies (Halliday & Goswami, 2013). Figure 4 also suggests that extinct fossil mammals had dental proportions remarkably similar to their extant relatives by the Oligocene. Two of the three fossil ursids (*Arctotherium* and *Ursavus*) fall within the MMC and PMM space of extant bears. Oligocene amphicynodont *Campylocynodon* and fossil ursid *Cyonarctos* are early carnivorans (Tomiya & Tseng, 2016) that fall directly within the MMC and PMM space of extant canids, further supporting the longevity of dental proportions in mammalian evolution and the association between these dental proportions and phylogenetic lineages. *Cyonarctos* is a fossil carnivoran in the subfamily Hemicyoninae from the Oligocene of Europe, noted for being a very “canid‐like” early ursid (de Bonis, 2013; Ginsburg & Morales, 1998). Based on the strong phylogenetic signal in dental proportions observed in the current study, and the clear distinction between dental proportions of extant ursids and canids, we suggest that either modern ursid dental proportions evolved relatively recently, or a reconsideration of the phylogenetic affinities of *Cyonarctos* and possibly also other hemicyonines may be warranted.

Our results also belie necessary caution when interpreting diet of fossil mammals exclusively from postcanine dental proportions (as captured by MMC and PMM), suggesting that other features of the dentition and skull, including individual tooth lengths relative to body size, are likely more useful for reconstructing diet. However, MMC and PMM dental proportions can play an important role in understanding the phylogenetic relatedness of extinct mammals, as these traits have strong phylogenetic signal in extant mammals. Our initial exploration of the fossil record also suggests that variation in mammalian dental proportions largely reflects bauplans that were established early in mammalian evolution and that are relatively stable over tens of millions of years. The fossil evidence supports our interpretation that there is significant phylogenetic constraint on the evolution of dental proportions within Boreoeutheria, as fossil mammals tend to have dental proportions similar to their extant counterparts. A larger assessment of the pattern of variation in dental proportions beyond Boreoeutheria to establish the “break points” in phylogenetic constraint will likely make MMC and PMM even more useful for assigning fossils to taxonomic groups.

In summary, we find that variation in postcanine dental proportions (as captured by MMC and PMM) accumulates slowly and characterizes mammalian lineages as they diversified from the plesiomorphic/ancestral ratios. Ancestral eutherian mammals had relatively homogeneous postcanine dental proportions, where the fourth premolar and all molars were similar in size (Butler & Clemens, 2001; Halliday & Goswami, 2013; Sloan & Van Valen, 1965; Ungar, 2010). From that homogeneous condition, several mammalian lineages diversified into distinct extant morphospaces that characterize the evolution of those groups (see Chiroptera, Canidae, Ursidae, and Suidae). Many other mammalian lineages have accumulated relatively little change and retain dental proportions that are similar to the ancestral condition (see Primates, Perissodactyla, Cervidae, and Chinchillidae). Whether this diversification results from the effects of genetic drift, genetic or developmental pleiotropy, and/or as‐of‐yet unidentified selective pressures, will be an essential question of future investigations.

## CONFLICT OF INTEREST

None declared.

## AUTHOR CONTRIBUTIONS

T.A.M. wrote the manuscript, T.A.M., J.‐R.B., M.F.B., S.M.C., R.D., S.R., A.S., R.T., S.Y., M.Z., and M.E.Z. collected the data. C.A.S. and P.S.U. contributed to the research idea and framework. L.J.H. directed the larger project in which this work was done and helped write the manuscript. All authors edited the manuscript and contributed to the intellectual content, context, and interpretation of this study.

## Data Availability

All raw data are available in the Supporting Information.
