# Supplementary material for: Evidence of strong stabilizing effects on the evolution of boreoeutherian (Mammalia) dental proportions
Source: Ecol Evol. 2019 Jun 14;9(13):7597–612. doi: 10.1002/ece3.5309 (PMC6635932; doi:10.1002/ece3.5309)
Supplement: Supplementary file 1 [file ECE3-9-7597-s001.docx]

**
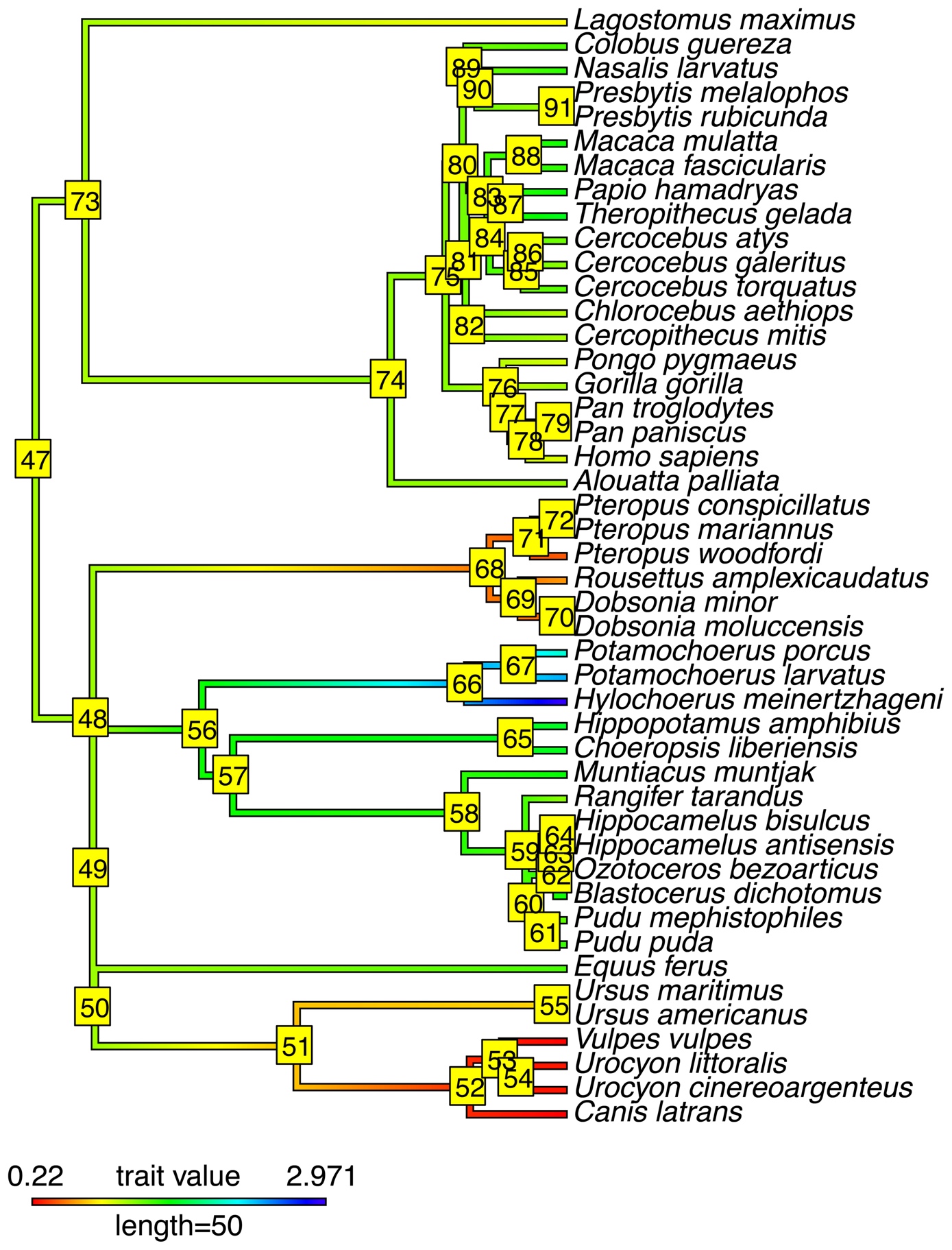
**

**Figure S1.** Ancestral state reconstruction of MMC values in Boreoeutheria with numbered nodes. See Table S5 for supported MMC values at each numbered node.


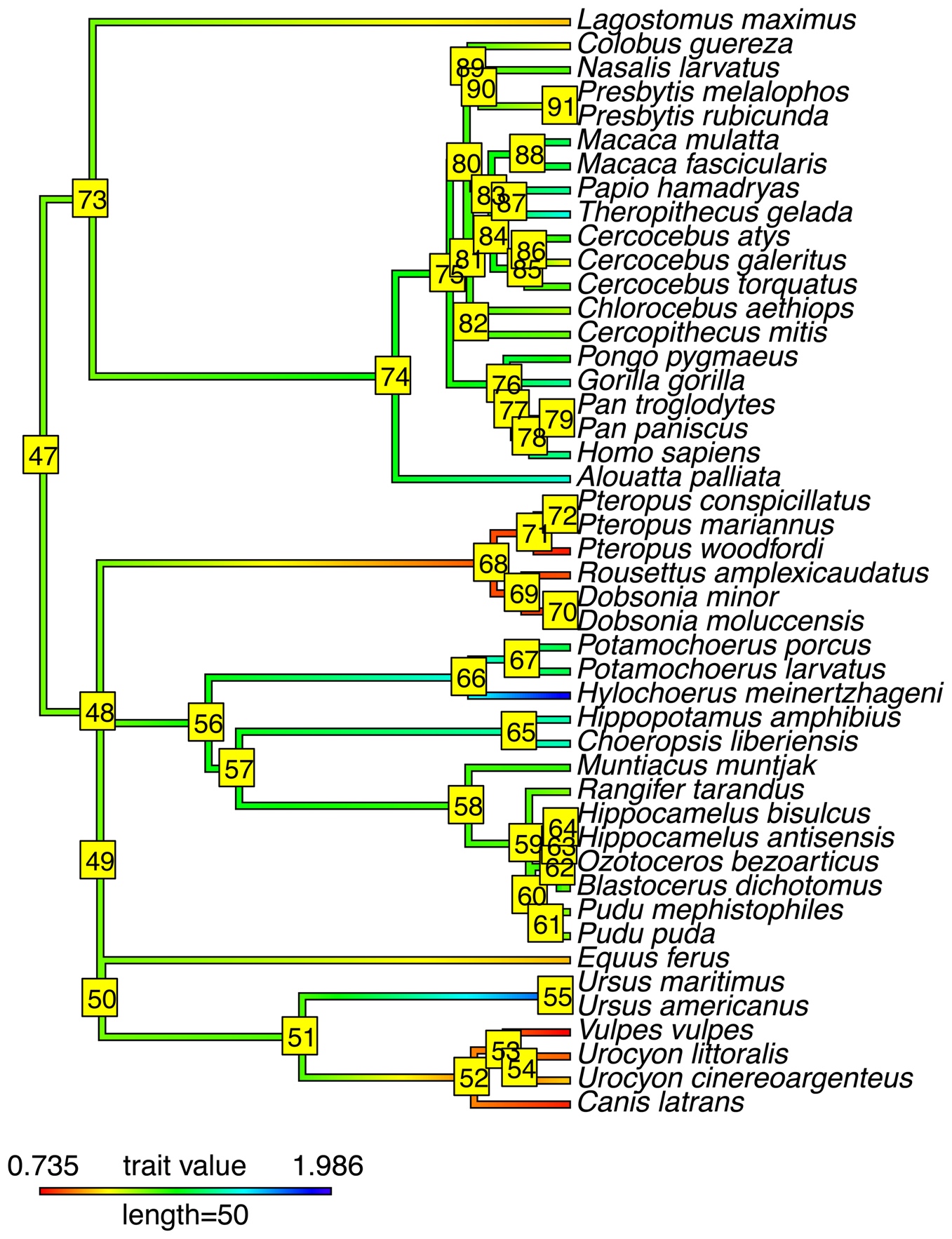


**Figure S2.** Ancestral state reconstruction of PMM values in Boreoeutheria with numbered nodes. See Table S5 for supported PMM values at each numbered node.


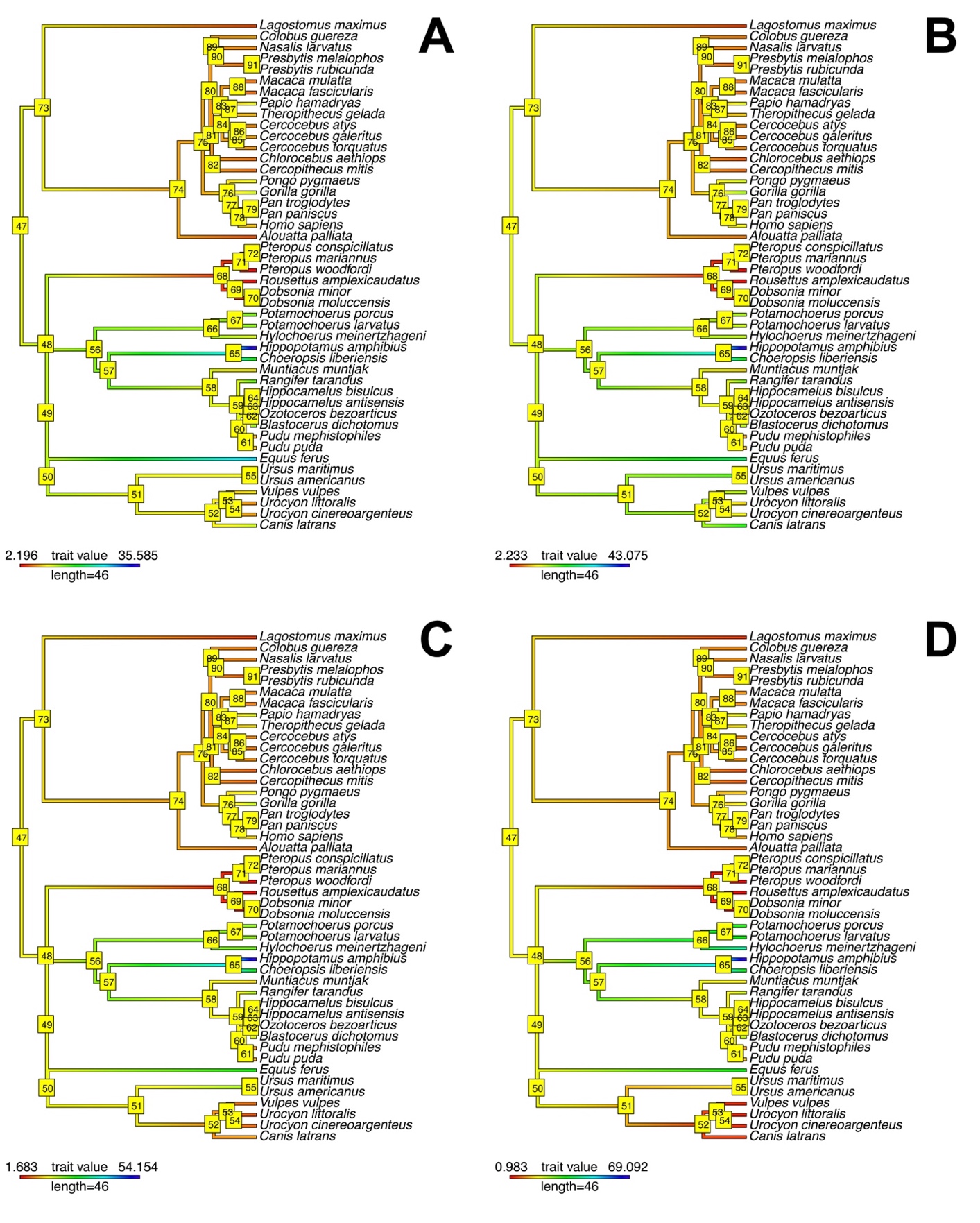


**Figure S3.** Ancestral state reconstruction of dental lengths for the (A) mandibular fourth premolar, (B) first molar, (C) second molar, and (D) third molar in Boreoeutheria, with numbered nodes. See Table S6 for supported dental lengths and calculated MMC and PMM at each numbered node.
